# Identification, expression and characterisation of a *Babesia bovis* hexose transporter

**DOI:** 10.1016/j.molbiopara.2008.06.010

**Published:** 2008-10

**Authors:** Elvira T. Derbyshire, Frits J. Franssen, Erik de Vries, Christophe Morin, Charles J. Woodrow, Sanjeev Krishna, Henry M. Staines

**Affiliations:** a Centre for Infection, Division of Cellular and Molecular Medicine, St. George's, University of London, Cranmer Terrace, London SW17 0RE, UK; b Virology Division, Department of Infectious Diseases and Immunology, Utrecht University, P.O. Box 80.165, NL-3508 TD Utrecht, The Netherlands; c Département de Chimie Moléculaire (UMR 5250, ICMG FR-2607, CNRS), 301 rue de la chimie, BP-53, 38041 Grenoble Cedex 9, France

**Keywords:** BboHT1/2, *Babesia bovis* hexose transporter 1/2, PfHT, *Plasmodium falciparum* hexose transporter, GLUT1, human facilitative glucose transporter 1, PCR, polymerase chain reaction, PBS, phosphate-buffered saline, Compound 3361, 3-*O*-(undec-10-en)-yl-d-glucose, Glucose, Transport, GLUT1, PfHT, Babesiosis, Oocyte

## Abstract

*Babesia* are tick-transmitted haemoprotozoan parasites that infect cattle, with an estimated 500 million at risk worldwide. Here, two predicted hexose transporters (*BboHT1* and *2*) have been identified within the *Babesia bovis* genome. BboHT1, having 40% and 47% amino acid sequence similarity compared with the human (GLUT1) and *Plasmodium falciparum* (PfHT) hexose transporters, respectively, is the only one that could be characterised functionally after expression in *Xenopus laevis* oocytes. Radiotracer studies on BboHT1 showed that it is a saturable, Na^+^-independent, stereo-specific hexose transporter, with a *K*_m_ value for glucose of 0.84 ± 0.54 mM (mean ± SEM). Using d-glucose analogues, hydroxyl positions at *O*-4 and *O*-6 have been identified as important for ligand binding to BboHT1. d-Glucose transport was inhibited maximally by cytochalasin B (50 μM). A long-chain *O*-3 hexose derivative (compound 3361) that selectively inhibits PfHT also inhibited relatively potently BboHT1, with an apparent *K*_i_ value of 4.1 ± 0.9 μM (mean ± SEM). Compound 3361 did not inhibit *B. bovis* proliferation in *in vitro* growth assays but inhibited invasion of glucose-depleted bovine erythrocytes. Taken together with results of inhibition studies with cytochalasin B and β-glucogallin, these data provide new insights into glucose metabolism of erythrocytic-stage *Babesia* infections.

## 1 Introduction

*Babesia* are tick-borne haemoprotozoan parasites, that cause severe and sometimes fatal infections in cattle, dogs and other mammals. *Babesia bovis* infects cattle in tropical and sub-tropical areas and exerts a high economic burden in regions that can least afford it [1,2]. *Babesia* parasites share many similarities, including their asexual blood-stage niche with other single-celled parasites such as *Plasmodia*, although comparative studies of these two parasites are few.

The membrane transporters of disease-causing, eukaryotic, unicellular parasites are being examined as targets for chemotherapeutic agents. In particular, glucose transporters have been studied intensively in kinetoplastids and apicomplexan organisms including *Plasmodium* and *Toxoplasma* species [3]. Previous studies on three *Babesia* species have examined glucose uptake into intact infected erythrocytes [4,5]. *B. bovis* infected bovine erythrocytes are far more permeable to glucose than uninfected erythrocytes due to the activity of a novel non-saturable channel/pore-like mechanism [5]. However, little is known about the molecular nature of this pathway or of any other glucose transport pathways, which are active during the intra-erythrocytic phase of the *Babesia* parasite's life cycle.

A *Plasmodium falciparum* hexose transporter (PfHT) has been cloned and characterised by function [6]. Localised to the parasite plasma membrane, PfHT is a facilitative carrier protein, phylogenetically related to mammalian glucose transporters (known as GLUTs, which belong to the major facilitative superfamily of transport proteins). Furthermore, PfHT has been validated as a drug target [7].

The aims of this work were to (i) identify potential facilitative hexose transporters in the *B. bovis* genome, (ii) clone, express, and characterise functionally candidate hexose transporters, using the *Xenopus laevis* oocyte heterologous expression system, and (iii) assess *B. bovis* hexose transporters as possible drug targets.

## 2 Materials and methods

### 2.1 Materials

Cytochalasin B, phloridzin, phloretin, and glucose analogues were obtained from Sigma–Aldrich (Dorset, UK). 3-*O*-(undec-10-en)-yl-d-glucose (compound 3361) and β-glucogallin were prepared, as described previously [8,9]. Radioisotopes ([^14^C]d-glucose and [^14^C]d-fructose) were obtained from Amersham (Bucks, UK). Inhibitors were added to cell suspensions as stock solutions in dimethyl sulphoxide.

### 2.2 Identification and cloning of *Babesia hexose* transporter sequences

Sequence data for *B. bovis* and *B. bigemina* were obtained from two sources: the *Babesia bovis* Genome Sequencing Project at http://www.vetmed.wsu.edu/research_vmp/babesia-bovis (Texas T2Bo strain) and The Wellcome Trust Sanger Institute at http://www.sanger.ac.uk (*B. bovis* Israel strain and *B. bigemina* Australian isolate). The latter sequence data were produced by the *Babesia bovis* EST Sequencing Group and the *Babesia bigemina* Sequencing Group at the Sanger Institute and can be obtained from ftp://ftp.sanger.ac.uk/pub/pathogens/babesia and ftp://ftp.sanger.ac.uk/pub/pathogens/Babesia/bigemina, respectively.

Using TBLASTN searches, two putative glucose transporter open reading frames (ORFs) were identified in the Texas strain genome (and partially identified in the Israel strain genome), on account of having significant homology with the published sequences for PfHT and GLUT1. These were termed *B. bovis* hexose transporter 1 (*BboHT1*; GeneBank™ accession number EU239929) and 2 (*BboHT2*; GeneBank™ accession number EU239930). For the Texas strain, the two ORFs were continuous, in the same orientation on chromosome 1 and separated by just over 1000 nucleotides.

Polymerase chain reactions (PCR) on genomic DNA from *B. bovis* Israel isolate (clonal line C61411), using AccuPrime *Taq* polymerase, was carried out with primers designed to introduce BglII restriction sites and a strong eukaryotic Kozak consensus sequence in front of the initiation codon (CACC*ATG*). Due to the incomplete sequence coverage of the Israel isolate, the Texas strain genome was used to generate primers when appropriate. Due to differences between the two genomes (see Section 4), care was taken to ensure that the synthetic primer sequences designed, using the Texas genome, did not introduce sequences from the Texas strain into the PCR products (by sequencing PCR products that independently covered these regions) or, if they did, that any changes were synonymous.

The resulting products were subcloned into BglII sites in the oocyte expression vector pSP64-T, which contains 5′- and 3′-untranslated *Xenopus laevis* β-globin sequences. The final product was verified by sequence analysis. Capped cRNA was then transcribed *in vitro* from Xba1 and EcoRI linearised *BboHT1* and *BboHT2* plasmids, respectively, using SP6 RNA polymerase (MEGAscript™ SP6, Ambion, Texas, USA) according to the manufacturer's protocol.

### 2.3 Expression in *Xenopus* oocytes and functional studies

*X. laevis* oocytes were prepared and used in transport studies as previously described [10]. Briefly, oocytes were harvested and connective tissue removed with collagenase treatment (2 mg/ml) for 2 h, while shaking. Oocytes were maintained at 18 °C in modified Barth's medium supplemented with 10 mg/l penicillin–streptomysin mix (Sigma–Aldrich, Dorset, UK). On the following day, stage V–VI oocytes were selected and microinjected with cRNA (20–40 ng in 30 nl of water) encoding *BboHT1/2* template or with a comparable amount of diethylpyrocarbonate-treated water. The oocytes were used for transport studies 48–72 h after cRNA injection.

Transport measurements were performed at room temperature, unless stated otherwise, on groups of 6–8 oocytes in Barth's medium containing, unless stated otherwise, either 38 μM d-glucose (3 μM radio-labelled and 35 μM unlabelled) or 100 μM d-fructose (2 μM radio-labelled and 98 μM unlabelled), and varying amounts of modulators, when required. After initial time-courses were performed (see Section 3), transport was measured over 10 min and corrected for uptake into paired, water-injected controls. The latter was performed to correct transport estimations for endogenous uptake, which can vary significantly between separate sets of oocytes (up to five-fold in the experiments reported here). All uptakes were linear for the times used in these assays and each result was confirmed by at least three independent experiments.

### 2.4 *B. bovisin vitro* cultivation and invasion assays

*B. bovis* (Israel strain, clonal line C61411 [11]) was cultured *in vitro* in bovine erythrocytes in 24-well plates (1.2 ml total volume) or in 25 cm^2^ bottles (15 ml volume) as described previously [12]. Cultures were grown in M199 medium supplemented with 40% adult bovine serum from a selected donor and 25 mM sodium bicarbonate at a packed cell volume of 5% at 37 °C, under an atmosphere of 5% CO_2_ in air. The parasitaemia of the *in vitro* culture was kept between 0.3% and 8% by dilution daily.

*B. bovis* *in vitro* invasion assays into normal and glucose-depleted bovine erythrocytes were performed, using free merozoites liberated from their host cells by electropulsing, as described previously [12]. Bovine erythrocytes were depleted of glucose by two washes in 10 volumes of VyMs buffer (a special solution used to maintain bovine erythrocytes in good condition during storage [13]) without glucose followed by 16 h of storage in VyMs without glucose. Prior to experimentation two additional washes were performed.

## 3 Results

### 3.1 Sequence analysis

Using sequence homology to PfHT, two putative *Babesia bovis* hexose transporter sequences (*BboHT1* and *BboHT2*) were identified and cloned from an Israeli isolate (see Section 2). *BboHT1* and *BboHT2* encode polypeptides of 472 and 587 amino acid residues, respectively, with estimated sizes of 51 and 63 kDa. BboHT1 has 40% and 47% amino acid sequence similarity compared with GLUT1 and PfHT, respectively; BboHT2 has 31% and 37%. A comparison of amino acid sequences between BboHT1 and BboHT2 determined 46% similarity.

**Fig. 1:**
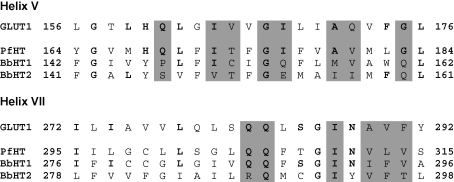
Alignments of transmembrane helices V and VII from GLUT1 with PfHT, BboHT1 (BbHT1) and BboHT2 (BbHT2). Bold letters indicate conserved residues compared with GLUT1 and shading indicates residues at the exofacial binding site of GLUT1, defined as being accessible to *p*-chloromercuribenzene-sulphonic acid if mutated to a cysteine residue.

As with other hexose transporters, both BboHT1 and BboHT2 are predicted to have 12 membrane spanning regions, using hydropathy plot analysis (with the SOSUI tool at bp.nuap.nagoya-u.ac.jp/sosui). In order to look for residues of functional importance, predicted membrane-spanning sequences for BboHT1/2 and PfHT were aligned with those of GLUT1 and residues shown in mutagenesis experiments to be of significance in the GLUT1 exofacial binding site examined specifically [14]. Of the 37 GLUT1 residues identified in this way, 13 were identical to aligned residues in PfHT, 10 were identical to aligned residues in BboHT1, and 9 were identical to aligned residues in BboHT2. An alignment of transmembrane helices V and VII is shown as an example (Fig. 1).

### 3.2 Transport kinetics

Radiotracer assays, using the *Xenopus laevis* oocyte heterologous expression system, were performed to assess if either BboHT1 or BboHT2 are facilitative glucose transporters. Over a 1 h period at room temperature, the uptake of d-glucose (38 μM external concentration) in oocytes injected with BboHT2 mRNA was 2.5 ± 1.0 pmol/oocyte, while the uptake in water-injected control oocytes was 3.6 ± 1.7 pmol/oocyte (mean ± SEM; *n* = 3; *P* = 0.6; unpaired, two-tailed Student's *t*-test). In addition, no differences in uptake rates were found between control and BboHT2 mRNA-injected oocytes for d-sorbitol, d-fructose and myo-inositol (data not shown).

Oocytes injected with BboHT1 mRNA demonstrated a large (typically 5–10-fold) increase in d-glucose uptake when compared with water-injected controls, 2–3 days after injection. Experiments at room temperature with 38 μM d-glucose, demonstrated linear uptake kinetics over 1 h (data not shown). The mean uptake of d-glucose after 1 h in oocytes injected with BboHT1 mRNA was 58 ± 12 pmol/oocyte, compared with an uptake of 8.8 ± 1.9 pmol/oocyte (mean ± SEM; *n* = 3) in water-injected controls. In the presence of 10 mM d-glucose linear uptake kinetics were lost after approximately 15 min (data not shown) so a 10 min time period was used for influx assays.

d-Fructose uptake also increased in oocytes expressing BboHT1 when compared with uptake in water-injected controls, having linear kinetics over 1 h (100 μM d-fructose at room temperature (data not shown)), and a mean uptake value of 44 ± 7 pmol/oocyte (compared with 15 ± 4 pmol/oocyte in water-injected controls; mean ± SEM; *n* = 3). These data suggest that d-glucose transport via BboHT1 is approximately four times faster than d-fructose (at relatively low, non-saturating concentrations).

**Fig. 2:**
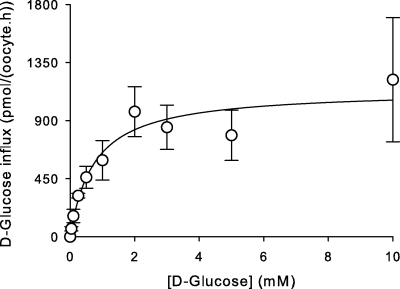
Concentration-dependence of the influx of d-glucose in oocytes injected with BboHT1 mRNA. All values presented were first corrected for the uptake of d-glucose into water-injected controls. Data are averaged from three experiments, each on oocytes from a different toad, and are shown as means ± SEM.

Fig. 2 shows the concentration-dependence of d-glucose influx in oocytes injected with BboHT1 mRNA. Data are consistent with a saturable system that conforms to Michaelis–Menten kinetics, with values for *K*_m_ of 0.84 ± 0.54 mM and *V*_max_ of 1181 ± 501 pmol/(oocyte.h) (mean ± SEM; *n* = 3).

### 3.3 Temperature and pH

**Fig. 3:**
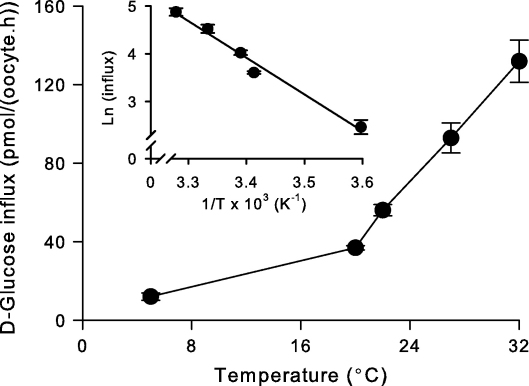
Temperature-dependence of the influx of d-glucose in oocytes injected with BboHT1 mRNA. Inset: Arrhenius plot constructed from the data in the main figure. All values presented were first corrected for the uptake of d-glucose into water-injected controls. The extracellular d-glucose concentration was 38 μM. Data are averaged from three experiments, each on oocytes from a different toad, and are shown as means ± SEM.

The effect of temperature (5–32 °C) on the transport of d-glucose via BboHT1 is shown in Fig. 3. An Arrhenius plot (Fig. 3 Inset) revealed the energy of activation (*E*_a_) to be 64 ± 3 kJ mol^−1^ or 15 ± 1 kcal mol^−1^ (mean ± SEM; *n* = 3). To test for the effect of pH, assays for d-glucose influx via BboHT1 over a range of pH values (5.5–9.0) were performed and no pH sensitivity was observed (data not shown).

### 3.4 Inhibitor studies

**Fig. 4:**
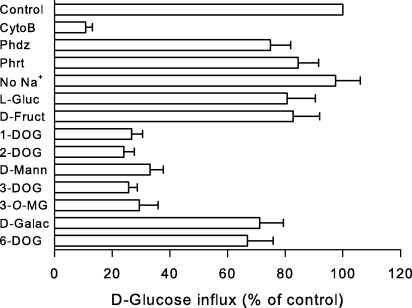
Effect of modulators on the influx of d-glucose in oocytes injected with BboHT1 mRNA. All values were first corrected for the uptake of d-glucose into water-injected controls and are presented as a percentage of paired experiments performed in the absence of any modulators. The extracellular d-glucose concentration was 38 μM. Data are averaged from three experiments, each on oocytes from a different toad, and are shown as means ± SEM. Control, influx in oocytes injected with BboHT1 mRNA in the absence of modulators; CytoB, cytochalasin B (50 μM); Phdz, phloridzin (50 μM); Phrt, phloretin (50 μM); No Na^+^, sodium-free Barth's medium where Na^+^ was replaced with equimolar choline chloride; l-Gluc, l-glucose (10 mM); d-Fruct, d-fructose (10 mM); 1-DOG, 1-deoxy-d-glucose (10 mM); 2-DOG, 2-deoxy-d-glucose (10 mM); d-Mann, d-mannitol (10 mM); 3-DOG; 3-deoxy-d-glucose (10 mM); 3-*O*-MG, 3-*O*-methyl-d-glucose (10 mM); d-Galac, d-galactose (10 mM); 6-DOG, 6-deoxy-d-glucose (10 mM).

Fig. 4 shows the effects of hexose transporter modulators on the influx of d-glucose (38 μM external concentration) mediated by BboHT1 in oocytes. Cytochalasin B (50 μM) inhibited d-glucose influx, but there was little inhibition by phloridzin and phloretin (both at 50 μM). Replacement of Na^+^ by either K^+^ or choline did not alter d-glucose transport. The presence of either l-glucose or d-fructose (both at 10 mM) in the medium reduced the influx of d-glucose by less than 20%.

The ligand interactions between BboHT1 and d-glucose were assessed, using d-glucose analogues (all at 10 mM concentrations). 1-Deoxy-d-glucose, 2-deoxy-d-glucose, d-mannose (the 2-epimer of glucose), 3-deoxy-d-glucose, and 3-*O*-methyl-d-glucose all reduced d-glucose uptake via BboHT1 by greater than 50% of control values. In contrast, d-galactose (the 4-epimer of glucose) or 6-deoxy-d-glucose competed relatively poorly with d-glucose uptake via BboHT1.

**Fig. 5:**
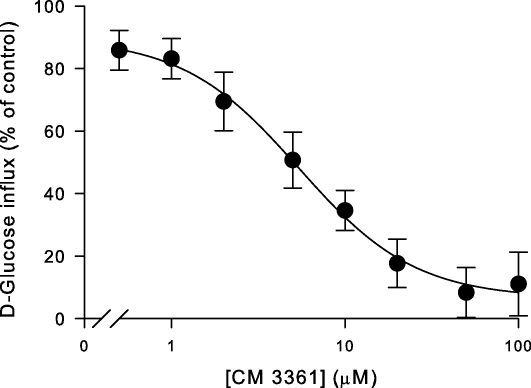
Effect of compound 3361 on the influx of d-glucose in oocytes injected with BboHT1 mRNA. All values were first corrected for the uptake of d-glucose into water-injected controls and are presented as a percentage of paired experiments performed in the absence of compound 3361. The extracellular d-glucose concentration was 38 μM. Data are averaged from four experiments, each on oocytes from a different toad, and are shown as means ± SEM.

An *O*-3 derivative of d-glucose was also examined in greater detail because it is a specific inhibitor of PfHT [7]. This 3-*O*-(undec-10-en-)-1-yl-d-glucose derivative, (compound 3361) has an apparent *K*_i_ value (the dissociation constant for inhibitor binding, which can be derived from the IC_50_ value, the concentration of inhibitor required to reduce transport by 50%, using the equation *K*_i_ = IC_50_/(1 + [substrate]/*K*_m_)) of approximately 50 μM for PfHT mediated glucose uptake. Fig. 5 shows the effect of compound 3361 on d-glucose transport via BboHT1. Compound 3361 inhibited d-glucose transport with an apparent *K*_i_ value of 4.1 ± 0.9 μM (mean ± SEM; *n* = 4). β-Glucogallin, which has anti-babesial properties [15], was tested to see if it inhibited d-glucose transport via BboHT1 but was ineffective at concentrations up to 100 μM (data not shown).

### 3.5 Growth and invasion assays

**Fig. 6:**
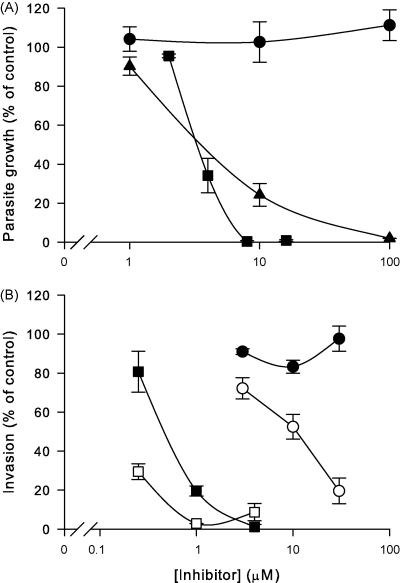
Effect of compound 3361 (circles), cytochalasin B (squares) and β-glucogallin (triangles) on the *in vitro* growth of *B. bovis* parasites (A) and *B. bovis* merozoite invasion into normal (closed symbols) and glucose-depleted (open symbols) bovine erythrocytes (B). For growth assays, *in vitro* cultures were established at an initial parasitaemia of 0.3% and the final parasitaemia was determined after 48 h of growth and expressed as a percentage of control experiments performed in the absence of inhibitors. For invasion assays, the parasitaemia was determined 2 h after initiating invasion and expressed as a percentage of control experiments performed in the absence of inhibitors (100% invasion normally represented a parasitaemia between 0.5% and 1% after 2 h). Data are averaged from three experiments and are shown as means ± SEM.

Compound 3361, cytochalasin B and β-glucogallin were tested in *in vitro* growth assays of *B. bovis* (Fig. 6A). Cytochalasin B and β-glucogallin inhibited completely *in vitro* growth at concentrations of 8 and 100 μM, respectively, with IC_50_ values (the concentration of inhibitor required to reduce parasite growth by 50%) between 1 and 10 μM. However, there was no effect of compound 3361 (up to 100 μM) on parasite growth.

Cytochalasin B inhibits *B. bovis* merozoite invasion of bovine erythrocytes [12] so invasion assays were also used to examine compound 3361, cytochalasin B and β-glucogallin (Fig. 6B). The assays, which use glucose-free media (see Section 2), were performed using both normal and glucose-depleted bovine erythrocytes (the latter having an invasion rate of approximately two thirds of the former). β-Glucogallin did not inhibit measurably invasion of normal or glucose-depleted bovine erythrocytes (data not shown), indicating that its effect on *in vitro* cultivation of *B. bovis* is due to inhibition of the intracellular growth phase. In contrast, cytochalasin B inhibited strongly parasite invasion into normal bovine erythrocytes, inhibiting well over half of the invasions measured in control experiments at a concentration of 1 μM. This effect was enhanced by the use of glucose-depleted bovine erythrocytes. Similarly, compound 3361 inhibited erythrocyte invasion in a glucose-dependent fashion, as invasion was blocked only into glucose-depleted bovine erythrocytes.

## 4 Discussion

This study has identified two putative *B. bovis* hexose transporters, using genome sequences for the Texas T2Bo and Israeli strains [16,17]. Significant inter-strain sequence differences are obvious when comparing nucleotide sequences for BboHT1 (95.8% nucleotide identity resulting in a predicted 97.7% conservation of amino acid sequence). BboHT2 is even more variable between strains with 83.1% of nucleotide conservation between the two *BboHT2* sequences (giving 83.8% conservation of amino acids). The functional significance of BboHT1 variability has not been examined, but does not alter any known functionally important residues (see below). Orthologues of BboHT1 and 2 were also found in the *B. bigemina* genome sequence (see supplementary file for alignments). However, while both BboHT1 and 2 had all the hallmarks of hexose transporters (discussed below), it was only possible to characterise functionally BboHT1 in *Xenopus* oocytes. There are several possible reasons for unsuccessful functional characterisation in heterologous expression systems [18]. Attempts to localise BboHT2 to the oocyte plasma membrane, with tagged (c-myc) versions, were inconclusive (data not shown) and so, in the absence of additional data to support its functional expression, it is not possible to comment further on the role of BboHT2.

In addition to the 12 predicted transmembrane helices and amino acid sequence similarities with GLUT1 and PfHT, BboHT1 (and BboHT2) contains many functionally important residues or motifs associated with facilitative hexose transporters. These include GRR/K motifs in the hydrophilic loops that connect transmembrane segments II and III and transmembrane segments VIII and IX, some, but not all, conserved residues involved in the exofacial binding of glucose (see Fig. 1), and a tryptophan residue in helix XI, which is involved in cytochalasin B binding [14]. Functionally, BboHT1 is a Na^+^-independent, cytochalasin B-sensitive, stereo-selective, saturable hexose pathway confirming that BboHT1 is a member of the facilitative sugar transporter family [19].

Both putative hexose transporter sequences are present in the *B. bovis* EST sequencing project. As the cDNA library used in this project was derived from infected erythrocyte cultures [17], this suggests that they are expressed during the erythrocytic phases of the parasite's life cycle. Unlike human erythrocytes, bovine erythrocytes have a naturally low permeability to d-glucose although infection by the *B. bovis* parasite increases the erythrocyte's permeability to d-glucose significantly [5]. This increase is via a channel/pore-like mechanism, which is characteristically neither stereo-specific nor saturable (unlike BboHT1). It could therefore be hypothesised that the intra-erythrocytic *B. bovis* parasite is able to obtain a supply of d-glucose from the plasma reservoir (maintained at 3.5 mM under normal conditions) via the novel channel/pore-like mechanism in sequence with BboHT1 on the parasite plasma membrane. Further experimentation is required to localise BboHT1 and characterise d-glucose transport across the parasite plasma membrane. However, experiments to determine the latter are technically challenging and will require the development of protocols to (i) remove/permeablise the host plasma membrane and (ii) dissect glucose transport from other factors such as metabolism, binding and experimental “noise”.

While the *B. bovis* genomes contain the majority of sequences for enzymes involved in oxidative metabolism [16,17], the lack of coding sequence for pyruvate dehyrogenase (required to produce acetyl CoA) and the low abundance of malate dehydrogenase (involved in the tricarboxylic acid cycle) from blood-stage parasite preparations [20] suggest that blood-stage *B. bovis* derives its energy primarily from glycolysis. Infected bovine erythrocytes consume significantly more d-glucose than uninfected erythrocytes [21]. This, coupled with the increased permeability of the host's plasma membrane to d-glucose [5], suggests that parasite survival is dependent on a supply of d-glucose. However, compound 3361, previously reported to kill *P. falciparum* by inhibition of PfHT [7], had no effect on *B. bovis* growth rates. This is somewhat unexpected given that compound 3361 is at least 10-fold more potent at inhibiting BboHT1 compared with PfHT when the two transporters are expressed in oocytes, a result that makes BboHT1 the most susceptible of hexose transporters tested to date [22].

There are several possible reasons for the lack of observed effect. Compound 3361 may not reach the intraerythrocytic site of BboHT1. Alternatively, another d-glucose transport pathway is present, which is insensitive to compound 3361 (for example BboHT2), or the parasite is not reliant completely on d-glucose as an energy source and is able to obtain energy from other substrates. With regard to the latter, one possibility is that parasites can oxidise glutamate via glutamate dehydrogenase (present in blood-stage preparations [20]) and feed the resulting α-ketoglutarate into the tricarboxylic acid cycle. Also, BboHT1 may have functional redundancy that requires near complete inhibition before affecting parasite growth. These data cannot validate BboHT1 as a drug target or distinguish between these possibilities.

Given that *B. bovis* invasion assays work in the absence of glucose-containing media, an interesting observation from this study is the ability of cytochalasin B and compound 3361 to inhibit parasite invasion via a mechanism(s) that is dependent on the host's cytosolic d-glucose concentration. One possible explanation for this observation is that the parasites do require d-glucose for invasion and are able to obtain it as it leaks from the uninfected erythrocytes (or obtain it directly as the parasites invade), although further studies would be required to confirm this hypothesis.

The number of apicomplexan parasite hexose transporters characterised functionally is steadily growing [6,23,24], allowing detailed comparison. The *K*_m_ value of BboHT1 for d-glucose is 0.84 mM, which is similar to the *K*_m_ values of the hexose transporters from the human malarial parasites, *P. falciparum* (PfHT; 0.97 mM), *P. vivax* (PvHT; 0.85 mM), and *P. knowlesi* (PkHT; 0.67 mM) but higher than those for the rodent malarial parasite, *P. yoelii* (PyHT; 0.12 mM) and the human pathogen, *Toxoplasma gondii* (TgHT; 0.03 mM). The relatively high *K*_m_ value (low affinity) of BboHT1 for d-glucose suggests that parasites may access a relatively rich supply of d-glucose or that they do not depend wholly on d-glucose for energy.

The low temperature-dependence of BboHT1 activity is similar to other apicomplexan hexose transporters [25]. This is consistent with the possible requirement of these transporters to function during parasite growth within invertebrate vectors (which live between 22 and 26 °C). The exception is the high temperature-dependence, *T. gondii* hexose transporter, consistent with the fact that *T. gondii* does not use an invertebrate vector. Like all other apicomplexan hexose transporters tested so far [6,25], BboHT1 is sensitive to cytochalasin B (consistent with the presence of a conserved tryptophan residue in helix XI, involved in cytochalasin binding [14]), is Na^+^-independent, pH-insensitive and is dependent on its substrates containing an *O*-4 hydroxyl group for high affinity interaction. However, it is the only apicomplexan hexose transporter, characterised so far, that is not dependent on its substrates containing an *O*-3 hydroxyl group (in the same way that PfHT is the only apicomplexan hexose transporter that is not dependent on its substrates containing an *O*-6 hydroxyl group [6]). This difference may help to identify residues or motifs that are involved in binding *O*-3 (and *O*-6) hydroxyl groups, which could help to explain the increased effect compound 3361 has upon BboHT1 when compared with other hexose transporters. These comparative studies may lead to improved rational drug design focusing on hexose transporters of parasites such as the *P. falciparum* orthologue that is validated as a drug target.

The ability of BboHT1 to transport fructose is also noteworthy. All the reported apicomplexan hexose transporters have been shown to transport fructose [24,25], even though the reason behind retaining this ability is not obvious for *Toxoplasma* and *Babesia* parasites. While able to transport d-fructose, BboHT1 has a lower affinity for d-fructose than for d-glucose (>10-fold), as suggested by the minor effect 10 mM d-fructose exerted on d-glucose influx (see Fig. 4). This is consistent with findings from PfHT, which has *K*_m_ values for d-glucose and d-fructose transport of approximately 1 and 12 mM, respectively [25]. Based on functional evidence for PfHT and sequence comparison for the other apicomplexan hexose transporters, including BboHT1, their ability to transport fructose does not involve the fructose selectivity filter found in helix VII of mammalian GLUTs [26]. Instead of this filter, a conserved glutamine residue in PfHT is important functionally for fructose transport [24,27]. However, BboHT1 does not contain this residue, raising the possibility that other novel sequences have evolved to allow fructose transport in *Babesia* parasites.

When taken with previous studies on mammalian GLUTs [23], other apicomplexan hexose transporters [6,24,25,27] and the hexose transporters of the kinetoplastidae [3,28], our studies increase understanding of how apicomplexan parasites adapt to different micro-environments. They also explore the possibility of targeting this class of transporter in different pathogens.
