# Supplementary material for: Identification, expression and characterisation of a Babesia bovis hexose transporter
Source: Mol Biochem Parasitol. 2008 Oct;161(2):124–9. doi: 10.1016/j.molbiopara.2008.06.010 (PMC2688680; doi:10.1016/j.molbiopara.2008.06.010)
Supplement: Supplementary file 1 [file mmc1.doc]

**Helix I**

BboHT1a ---------------------MRS**YFVYALALGALVAFNFGLNTVSF**VASREFAVMDLGW 39

BboHT1b ---------------------MRS**YFVYALALGALVAFNFGLNTVSF**VASREFAVMDLGW 39

BbiHT1 ---------------------MKPY**FACSVTLAAIVAFNFGLNTVNFN**ASKEFATVDFGW 39

BboHT2a ---------------------MSRYFW**AAMCCAGIVALNFGVIGCCLGPS**KMFAMRDMEV 39

BboHT2b ---------------------MSRYFW**AAMCCAGIVALNFGVIGCCLGPS**KLFAMREMRV 39

BbiHT2 ---------------------MAWYFWAAL**LAAGIVAFNFGVICVSLGPSKQF**AFKEMGI 39

PfHT MTKSSKDICSENEGKKNGKSGFFST**SFKYVLSACIASFIFGYQVSVLN**TIKNFIVVEFEW 60

hGLUT1 --------------MEPSSKK**LTGRLMLAVGGAVLGSLQFGYNT**GVINAPQKVIEEFYNQ 46

: : . : :: ** : . : .

**Helix II**

**Helix III**

BboHT1a CAGQ----DKLYECSRASTY**SDIIAAGTFIGGAVGSLLIGYFA**-KFGRRK**GMLIIHIINV** 94

BboHT1b CTGE----AKLYECSRASTY**SDIIAAGTFIGGAVGSLLIGYFA**-KFGRRK**GMLIIHIINV** 94

BbiHT1 CKGEG--NANEYECTKAT**LYGSLIGGGSFIGGILGSLLIGV**IS-KYGRRR**AMIIIHAINI** 96

BboHT2a YS-----GEQLPEGKKYK**FVDSLLSGSVFLGCALGSALISI**LS-KYGRH**KCMIIINLLSV** 93

BboHT2b YS-----GKQVDDSKKKT**FVDSLLSGSVFLGCALGSALISI**LS-KYGRH**KCMIIINLLSV** 93

BbiHT2 EEKRHCGGKQCSKDNN-E**LSRILISGAVFLGCAIGSICIGL**LS-HWGRRK**CMILIHLLSI** 97

PfHT CKGE----KDRLNCSNN**TIQSSFLLASVFIGAVLGCGFSG**YLV-QFGRR**LSLLIIYNFFF** 115

hGLUT1 TWVHR--YGESILPTTL**TTLWSLSVAIFSVGGMIGSFSVG**LFVNRFGRR**NSMLMMNLLAF** 104

. . : . :* :*. . : ::**: :::: : .

**Helix IV**

**Helix III**

**Helix V**

BboHT1a **IGSCMS**---**TSASCFT**MFLLG**RLIAGVSVGASGVVA**-**IYLSEICT**NESRG**KFGIVYPLFI** 150

BboHT1b **IGSCMS**---**TAASCFT**MFLLG**RLIAGVSVGASGVVA**-**IYLSEICT**NESRG**KFGIVYPLFI** 150

BbiHT1 **VGSLIS**---**TAAQCFS**MLLLGR**LIAGVSVGMSGMIA**-**IYLTEICTV**ENRGT**YGTAYPMFI** 152

BboHT2a **IGCFGA**---**SVGQ**HWSLF**MISRLLSGISVGMSGVAA**-**MYLSE**ICPRSKRG**MFGALYSVFV** 149

BboHT2b **VGCFGA**---**SVGG**HWSLF**MISRLLSGISVGMSGVAA**-**MYLSE**ICPRSKRG**MYGAIYSVFV** 149

BbiHT2 **AGCGVA**---**AIGKH**WQLFA**LARLSAGVSVGMSGVAS**-**LYLGEI**CSSDKR**GLYGALYSVFV** 153

PfHT **LVSILT**---**SITHHF**HT**ILFARLLSGFGIGLVTVSVPMYI**SEMTHKDKKGAY**GVMHQLFI** 172

hGLUT1 **VSAVLMGFSKLG**KSFEM**LILGRFIIGVYCGLTTGFVPMYV**GEVSPTAFRGA**LGTLHQLGI** 164

. : : :.*: *. * :*: *: :* * : : :

**Helix V**

BboHT1a **CIGQFLMVAWQLL**HGRILG----------------------------------------A 170

BboHT1b **CIGQFLMVAWQLL**HGRILG----------------------------------------A 170

BboHT2a **TFGEMAIIMFQLS**HGKNLD------------------------------------DSGKC 173

BboHT2b **TFGEMAIVMFQLS**HGDALN------------------------------------DSAKC 173

BbiHT2 **TLGELLISLWQL**SHAGSLDSLCTCDSCCCNKDCAERKCCCKDGKLCTNSENCCSCTGSNC 213

PfHT **TFGIFVAVMLGLAM**GEGPK-------------------------------ADSTEPLT-- 199

hGLUT1 **VVGILIAQVFGLDS**IMG------------------------------------------- 181

.* : *

**Helix VI**

BboHT1a DNKVVTMDDLK-------------------------TFDKFVWRM**AQFWPVVFSIIAVVI** 205

BboHT1b DGAAVTMDDLK-------------------------TFDKFVWRM**AQFWPVVFSIIAVVI** 205

BbiHT1 TENAAGADAIT-------------------------MMDRFVWRM**AQFWPAVFSMIALAI** 207

BboHT2a KCHFKKDVDIK---------------------------DKVLWR**ISQMAGGIFSLIGLYL** 206

BboHT2b KCHMPCKNQLK---------------------------DQILWR**ISQMAGGIFSLIGLYL** 206

BbiHT2 QCCCATKTEATKPVAARPEAAKTATAGCGGKLEADKSSDKAIWRM**GQLYGALASLVALCL** 273

PfHT ------------------------------------SFAKLWW**RLMFLFPSVISLIGILA** 223

hGLUT1 --------------------------------------NKDL**WPLLLSIIFIPALLQCIV** 203

: * : : :::

**Helix VI**

BboHT1a **MVFIVTDD**TPYVLLNEGKEEEARSVISKLHGEENADAITAELKADIEAQ-KANSDKLSLL 264

BboHT1b **MVFIVTDD**TPYVLLNEGKEEEARSVISKLHGEENADAITAELKADIEAQ-KANSDKLSLL 264

BbiHT1 **IYVVVKHD**TPHVLLQEGKEDEAKEVIELLHGSEKADTVFAEVRGDVEAL-QSASTSLGII 266

BboHT2a **LVTTVKE**DSPFILAKNGQLEEARHVLEMLQGKEHVEQSYNELMEDLASQSASGAKKASII 266

BboHT2b **LVTTVKE**DSPFVLAKNGQLEEARQVLEMLQGKQHVEQSYNELMEDLASQSASGAKKASIM 266

BbiHT2 **LFMVVVDD**TPFMLVKRGEHQKAKEVLALLQGPDKVESSFQEIMEDVASE-ESGAKKMGLW 332

PfHT **LVVFFK**EETPYFLFEKGRIEESKNILKKIYETDNVDEPLNAIKEAVEQNESAKKNSLSLL 283

hGLUT1 **LPFCP**-ESPRFLLINRNEENRAKSVLKKLRGTADVTHDLQEMKE--ESRQMMREKKVTIL 260

: ... ..* :... :.:: :: : .. : . :

**Helix VII**

**Helix VIII**

BboHT1a EALKVAQYRKVIF**ICCGLGIVQQFSGINIFVANAST**LFISIMGRTVI**ANVMGLVGILCLM** 324

BboHT1b EALKVAQYRKVIF**ICCGLGIVQQFSGINIFVANAST**LFISIMGRTVI**ANVMGLIGILCLM** 324

BbiHT1 AALKVPKYRRAIFIICGLSIM**QQLSGINVFVANASKLFVSIMGR**SFTA**SAMSLAGVAALF** 326

BboHT2a DAFKHPDSRYAL**FVVFGIAILRQMCGIYVFTLSAS**ELFGQIVGEGLKA**SAWGVLCPVVNF** 326

BboHT2b DAFKHPDSRYAL**FVVFGIAILRQMCGIYVFTLSAS**ELFGQIVGEGFKA**SAWGVLCPVANF** 326

BbiHT2 EALSYPDSRR**ALLVVFAIAILRQLCGIYVFTLS**ASEMFGMIVG**QGLGATAMGNLCPLTNF** 392

PfHT SALKIPSYRYVI**ILGCLLSGLQQFTGINVLVSNSN**ELYKEFLDSH**LIT**-**ILSVVMTAVNF** 342

hGLUT1 ELFRSPAYRQ**PILIAVVLQLSQQLSGINAVFYY**STSIFEKAGVQQPV**YATIGSG**--**IVNT** 318

: . * ::: : :*: ** . :. :: .

**Helix VIII**

**Helix IX**

BboHT1a **VTMISLSFFI**EKFGRKTL**LLAGIFVSSVFMVPAVIIKLAAG**------------------- 365

BboHT1b **ITMISLSFFI**EKFGRKTL**LLAGILVSSVFMVPAVIIKIAAG**------------------- 365

BbiHT1 **VTTLFLAFVID**KFGRKT**LFLFGIAVSCVSMVPAVIIKMSS**-------------------- 366

BboHT2a **LVCCTMPFYVE**KLGRR**TLLIIGSGSGFVIMAIALIMYA**THNNENNKNKKCKCCCCCCCSC 386

BboHT2b **LVCCAMPFYVE**KLGRR**TLFIIGSGSGFIIMAIALIIQA**TDGKGAEAKKNGDKCCCCSAES 386

BbiHT2 **IVCCLL**PLYIDKFGRRK**LLIYGSGIGTSVLAVCLLLNTL**CGDGEE--------------- 437

PfHT **LMTFPAIYI**VEKLGRK**TLLLWGCVGVLVAYLPTAIANEI**NR------------------- 383

hGLUT1 **AFTVVSLFVVER**AGRR**TLHLIGLAGMAGCAILMTIALAL**LE------------------- 359

::: **:.* : * :

BboHT1a -----------------------------------------------------------K 366

BboHT1b -----------------------------------------------------------K 366

BbiHT1 ------------------------------------------------------------

BboHT2a SPTSGGVCDSCTTSGHDGKCCCASCKTSSSKCSGGCNCSSGTGCSTSDTECSLLKNVEGK 446

BboHT2b GSSSGKCCTGGACK--NGGKSCACSKPEHPNCEP-------PSENEHHGECSFSKTTHG- 436

BbiHT2 ---SSNSCRKDSKK---------------------------------------------- 448

PfHT ------------------------------------------------------------

hGLUT1 ------------------------------------------------------------

**Helix XI**

**Helix X**

BboHT1a DAKWAEYIM**VTGCMGFMVGFAIGLGGVMWLYL**AEAMGAEYKDAG**FGLATCANWLFAAIVV** 426

BboHT1b EAKWAEYIM**VTGCMGFMVGFAIGLGGVMWLYL**AEAMGAEYKDAG**FGLATCANWLFAAIVV** 426

BbiHT1 AEKWASYVMV**VGCIGFMIGFAIGFGGIMWLYFA**EALGTEYKDA**AFGVATSVNWLFAAIVV** 426

BboHT2a SADWR**QYVMIVACILFVGCFATGYGGVS**WLYFGEALPPEYKDSA**YSVASILNWLTCAIVV** 506

BboHT2b DAKW**QKYFMIVACILFVGCFATSYGGVS**WLYFGEALPPEYKDSA**YSVASVLNWLTCAIVV** 496

BbiHT2 EARWP**QYLMIFGCMVFVAAFATGYGGVA**WLYFSEALGAEYRDA**GFAVASALNWLAAALVV** 508

PfHT NSNFVKI**LSIVATFVMIISFAVSYGPVLWI**YLHEMFPSEIKD**SAASLASLVNWVCAIIVV** 443

hGLUT1 QL**PWMSYLSIVAIFGFVAFFEVGPG**PIPWFIVAELFSQGPRP**AAIAVAGFSNWTSNFIVG** 419

: . . : . : :: * . * : *: . * : : :. .:* ** :*

**Helix XI**

**Helix XII**

BboHT1a **MTSGMLI**KFNENF**TYALYAGFGVLGFVYVALLIKET**KGIPLGQAYA-------------- 472

BboHT1b **MTSGMLI**KFNENF**TYALYAGFGVLGFVYVTFLIKET**KDIPLGQAYA-------------- 472

BbiHT1 **ISSDP**LLAWNDKV**AYSLYTGFGLLGFAFVYFFLKET**KGMPLGQAFA-------------- 472

BboHT2a **STAAPMQ**NALGD**KVYWFYVICSGIGCVFAILFVKE**TAGVPLGQAYKGATCPEIITKIASL 566

BboHT2b **STAKPMQ**NALSE**NVYWFYVICSGIGCVFAILFVKE**TAGVPLGQAYKGATCPEIITKIASL 556

BbiHT2 **MTADK**LRSVLSKN**VYWVYVFFSFISCLFAIFFVRET**KGVPLGQAYIGGGSPWSMRGTPEP 568

PfHT **FPSDI**IIKKSPS**ILFIVFSVMSILTFFFIFFFIKE**TKGGEIGTSPYITMEERQKHMTKSV 503

hGLUT1 **MCFQY**VEQLC**GPYVFIIFTVLLVLFFIFTYFKV**PETKGRTFDEIASGFRQGGASQSDKTP 479

: : .: : : : : ** . :.

BboHT1a ---------------------

BboHT1b ---------------------

BbiHT1 ---------------------

BboHT2a FTKTKPVIHEAAKTGMKNIRA 587

BboHT2b FTKTKPVIHEAAKTGMKNIRA 577

BbiHT2 DPYDWPHV------------- 576

PfHT ---------------------

hGLUT1 EELFHPLGADSQV-------- 492

Fig. S1. Sequence alignment and transmembrane predictions for hexose transporters. Six *Babesia* transporters, PfHT, and human GLUT1 are shown. The Clustal W program was used to generate the alignment. The residues highlighted in bold correspond to transmembrane segment predictions determined with the SOSUI program. Coloured sections highlight residues of interest (GRR/K motifs in positions characteristic of this superfamily of transporters (red) and a tryptophan residue in helix XI that is involved in binding to cytochalasin B (turquoise)). *BboHT1a, B. bovis* transporter 1, Israel isolate (Accession number EU239929); *BboHT1b, B. bovis* transporter 1, Texas isolate; *BboHT2a, B. bovis* transporter 2, Israel isolate (Accession number EU239930); *BboHT2b, B. bovis* transporter 2, Texas isolate; *BbiHT1, B. bigemina* transporter 1; *BbiHT2, B. bigemina* transporter 2; *PfHT, P. falciparum* transporter (AAJ131457); hGLUT1, human GLUT1 (AAK01395). *Asterisk*, identical or conserved residues in all sequences; *colon*, conserved substitutions; *dot*, semi-conserved substitutions.
